# In-Bath 3D Printing of Anisotropic Shape-Memory Cryogels Functionalized with Bone-Bioactive Nanoparticles

**DOI:** 10.1021/acsami.3c18290

**Published:** 2024-04-09

**Authors:** Edgar J. Castanheira, Luís P. G. Monteiro, Vítor M. Gaspar, Tiago R. Correia, João M. M. Rodrigues, João F. Mano

**Affiliations:** CICECO − Aveiro Institute of Materials, Department of Chemistry, University of Aveiro, 3810-193 Aveiro, portugal

**Keywords:** cryogels, nanocomposites, suspension 3D printing, directional freezing, shape memory

## Abstract

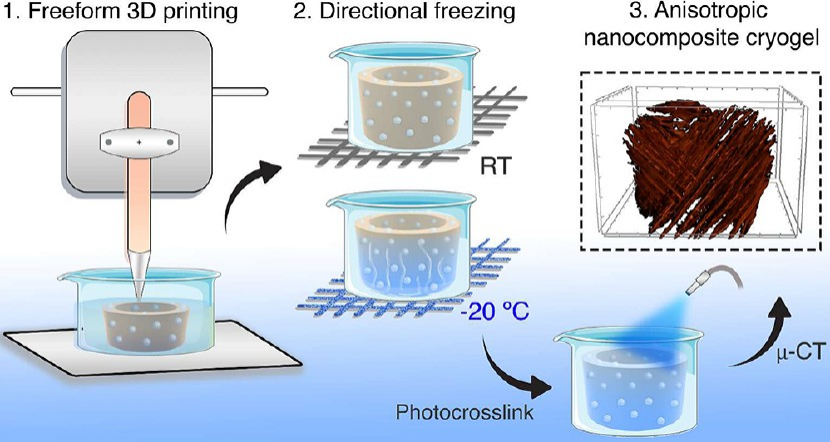

Cryogels exhibit unique shape memory with full recovery and structural stability features after multiple injections. These constructs also possess enhanced cell permeability and nutrient diffusion when compared to typical bulk hydrogels. Volumetric processing of cryogels functionalized with nanosized units has potential to widen their biomedical applications, however this has remained challenging and relatively underexplored. In this study, we report a novel methodology that combines suspension 3D printing with directional freezing for the fabrication of nanocomposite cryogels with configurable anisotropy. When compared to conventional bulk or freeze-dried hydrogels, nanocomposite cryogel formulations exhibit excellent shape recovery (>95%) and higher pore connectivity. Suspension printing, assisted with a prechilled metal grid, was optimized to induce anisotropy. The addition of calcium- and phosphate-doped mesoporous silica nanoparticles into the cryogel matrix enhanced bioactivity toward orthopedic applications without hindering the printing process. Notably, the nanocomposite 3D printed cryogels exhibit injectable shape memory while also featuring a lamellar topography. The fabrication of these constructs was highly reproducible and exhibited potential for a cell-delivery injectable cryogel with no cytotoxicity to human-derived adipose stem cells. Hence, in this work, it was possible to combine a gravity defying 3D printed methodology with injectable and controlled anisotropic macroporous structures containing bioactive nanoparticles. This methodology ameliorates highly tunable injectable 3D printed anisotropic nanocomposite cryogels with a user-programmable degree of structural complexity.

## Introduction

Polymeric hydrogels find widespread use in the biomedical field, particularly in tissue engineering,^1,2^ drug delivery,^3,4^ and wound healing,^5,6^ as they showcase important features such as high-water content and similarity to soft tissues.^7,8^ Despite their functionalities, hydrogels have drawbacks resulting from their 3D nanoporous network, leading to limited cell infiltration and nutrient diffusion, and resulting in suboptimal cell densities within the hydrogel matrix.^9^ Additionally, many efforts have been made to develop injectable hydrogels as an alternative to invasive surgical techniques.^10,11^ This strategy can be implemented as a bulk biomaterial or by using liquid precursors. However, limitations related to the injection of liquid precursors and the numerous prerequisites for cross-linking, such as (i) gelation time, (ii) homogeneity, (iii) cytocompatibility of the precursors, and (iv) cross-linking accessibility, limit the efficiency of the procedure.^12^ Cryogels represent a valuable alternative to bulk hydrogels, as they offer enhanced mechanical stability, deformability, and interconnected porosity, as well as suitable mass transport and cell permeability features.^13,14^ These structures are commonly fabricated by cross-linking under subzero temperatures to create a highly interconnected macroporous network. Upon thawing, the scaffold is able to maintain structural integrity under controlled compression forces.^15,16^ Additionally, the cooling of the structure can be controlled as a directional freezing process. Accordingly, by inducing a freezing front from the bottom to the top of the structure, the ice crystal orientation can be controlled and arranged in an organized ice template. Anisotropic 3D models with highly defined topography have proven successful in guiding cell migration and in supporting proliferation and differentiation.^17−20^ For instance, in collagen-rich tissues, such as bone, the alignment of the collage fibers is of extremely importance to preserve resistance to mechanical loading.^21,22^ Bone tissue can be described as a composite elastic matrix, comprising mostly collagen fibers, binding a major inorganic moiety of nanosized hydroxyapatite.^23^ Mesoporous silica nanoparticles (MSNPs), recognized for being osteoinductive/osteoconductive, biocompatible, and biodegradable, have been widely used to improve bone regeneration.^24−27^ They are known for their high pore volume, surface area to volume ratio, and the ability to be easily functionalized with different chemical moieties or ions.^28,29^ When doped with calcium and phosphate ions, MSNP’s bioactivity is tremendously enhanced toward the formation of new bone tissue.^30,31^ Therefore, a soft matrix of a cryogel, combined with hydroxyapatite-like nanoparticles, can mimic the bone composition, enhancing both the structural and functional properties of the nanocomposite structure.

To date, there are a few reports on the development of 3D printed cryogels for bone tissue regeneration.^32^ However, none has combined 3D freeform printing with directional freezing or fully explored the formulation of nanocomposite cryogel precursor inks for fabricating nanoparticle-doped cryogel constructs. In this sense, freeform 3D printing has emerged as a widely used technique for fabricating constructs with spatially defined architectures.^33,34^ Optimizing the fabrication of cryogels using this technique unlocks the design of complex cryogels with gravity-defying spatial arrangements.

**Scheme 1:**
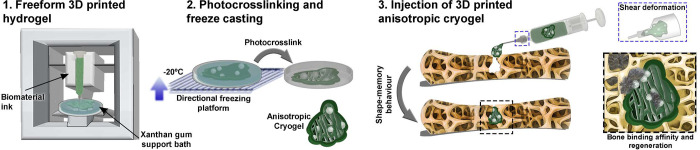
Overview of the Development of Injectable 3D Printed Anisotropic Nanocomposite Cryogels Combination of directional freezing with freeform 3D printing (i.e., using a xanthan gum support bath). Fabrication of macroporous cryogels of methacrylated laminarin (MetLAM) with mesoporous silica nanoparticles (MSNPs) doped with calcium and phosphate. The resultant scaffolds are injectable with shape-memory and osteogenic potential. Some elements were adapted from BioRender.com icons library.

In this study, nanocomposite anisotropic cryogels were developed for potential applications in the orthopedic field, mainly for bone tissue regeneration. Xanthan gum was employed as a support bath for freeform 3D printing, and directional freezing was incorporated to achieve anisotropy (Scheme 1). Freeform 3D printed cryogels exhibit a unique combination of features, including: (i) anisotropy, (ii) injectability, (iii) shape memory, and (iv) incorporation of bone-bioactive nanoparticles. To the best of our knowledge, this is the first time that 3D freeform printed anisotropic nanocomposite cryogels is reported.

## Results and Discussion

### Synthesis and Characterization of Precursor Compounds

**Figure 1:**
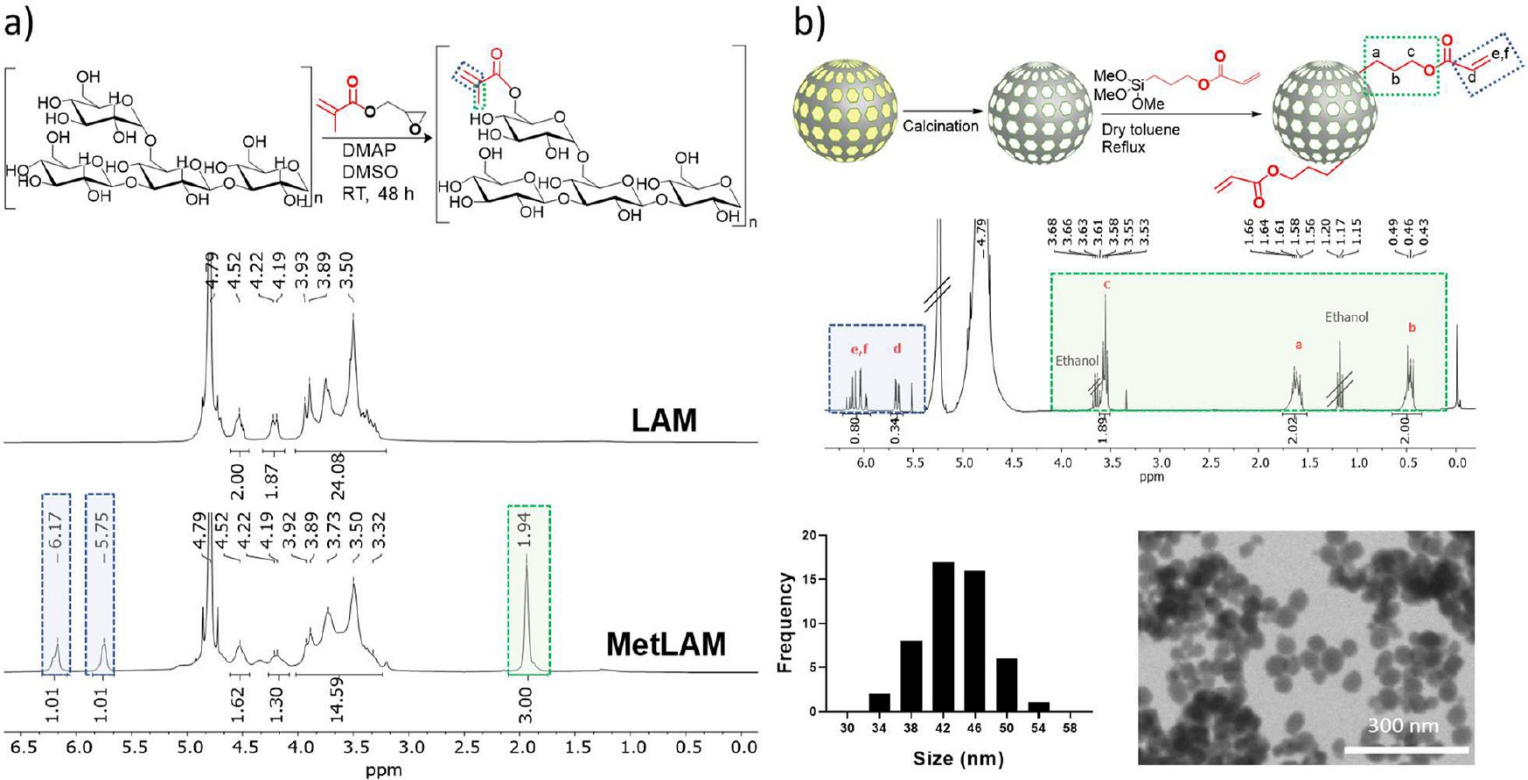
Design of hydrogel precursors. a) Reaction scheme of the synthesis of MetLAM and ^1^H NMR spectra of LAM (top) and MetLAM (bottom), in D_2_O. b) MSNPs-CaP illustration of the template removal (CTAB) followed by NP surface modification with acrylate-TMOS (top). ^1^H NMR spectra of modified MSNPs-CaP with TMOS-PA, in D_2_O at pH = 13 (center). TEM picture and analysis of unmodified MSNPs-CaP with the relative size histogram, *n* = 50 (bottom).

Laminarin (LAM) is a branched low-molecular-weight natural-origin polysaccharide found in brown algae, and due to its unique biological properties, it has been explored in the biomedical field.^35−38^ A previously optimized method was followed^39^ to introduce photopolymerizable methacryloyl groups in the LAM backbone (MetLAM). The precursor structure was characterized by proton nuclear magnetic resonance (^1^H NMR) (Figure 1a) and attenuated total reflectance–Fourier transform infrared (ATR-FTIR) spectroscopy (Figure SI1). ^1^H NMR confirmed the modification of LAM with the appearance of two signals of the vinylic protons (δ = 6.17 and δ = 5.75 ppm). The degree of substitution (DS) was determined by ^1^H NMR, through the ratio of the vinylic proton integral peak against the polymer backbone region (δ = 3.32–4.52 ppm), resulting in a DS of 10 ± 4%. A comparison of ATR-FTIR spectra confirmed MetLAM by the exhibition of characteristic peaks, consistent with the main vibrational modes of methacryloyl groups, including the C=O stretching (1720 cm^–1^).^35^

### Mesoporous Silica Nanoparticles (MSNPs) Synthesis and Characterization

MSNPs (MCM-41 based)^40−44^ are not able to form hydroxyl carbonate apatite; however, they can regulate osteoblasts by releasing Si ions, promoting the formation of new bone matrix. Moreover, when doped with Ca^2+^ (Ca) and PO_4_^3–^ (P) ions, MCM-41 MSNPs have shown a great potential in promoting hydroxyapatite mineralization.^45−47^ Herein, we optimized the formulation of MSNPs-CaP. Additionally, the MSNPs-CaP surface was also modified with acrylate photopolymerizable groups.^48^ Therefore, during the photo-cross-linking of the cryogel, the MSNPs bound directly onto MetLAM matrix (Figure 1b top). The CaP ratio was kept around 1.67 during the process and to an overall final content of 10% (w/w in relation to the CTAB synthesis precursor). Using this strategy, it was possible to avoid micelle disruption and consequent silica deposition. MSNPs-CaP were tailored for a particle size of 50 nm. The particles were hydrolyzed in a basic deuterated solution (pH = 13) and characterized by ^1^H NMR.^49^ It was possible to confirm (Figure 1b, center) the successful modification by the presence of the vinylic protons (δ = 6.06 and 5.66 ppm) and the proton peaks from the propyl chain (δ = 3.55, 1.61, and 0.47 ppm, respectively). Mesopore analysis was conducted using BET,^50,51^ and a pore size in the range of 4.7 to 5.3 nm was obtained (Figure SI2). The MSNPs-CaP diameter was determined through transmission electron microscopy (TEM) averaging 44 ± 4 nm, where it was also possible to observe a good nanoparticle distribution (Figure 1b, bottom). Furthermore, MSNPs-CaP size and distribution were also evaluated by dynamic light scattering (DLS), with MSNPs-CaP presenting 142 ± 4 nm of hydrodynamic diameter and a polydispersity index (PDI) of 0.13 (Figure SI3). The amounts of Ca and P, as well as their ratio, were quantified with scanning electron microscopy (SEM) coupled with energy dispersive X-ray spectroscopy (SEM-EDS). The ratio among the total Si, Ca, and P evidenced an average of 6.6% of Ca and P elements, in a one-to-one ratio (wt %) (Figure SI4), which is similar to ratios and weight percentage with previously synthesized MSNPs-CaP.^52^ The acrylate modified MSNPs doped with calcium and phosphate will be presented as MSNPs-CaP, since only acrylate modified MSNPs-CaP were used within this work.

### Cryogel Fabrication

**Figure 2:**
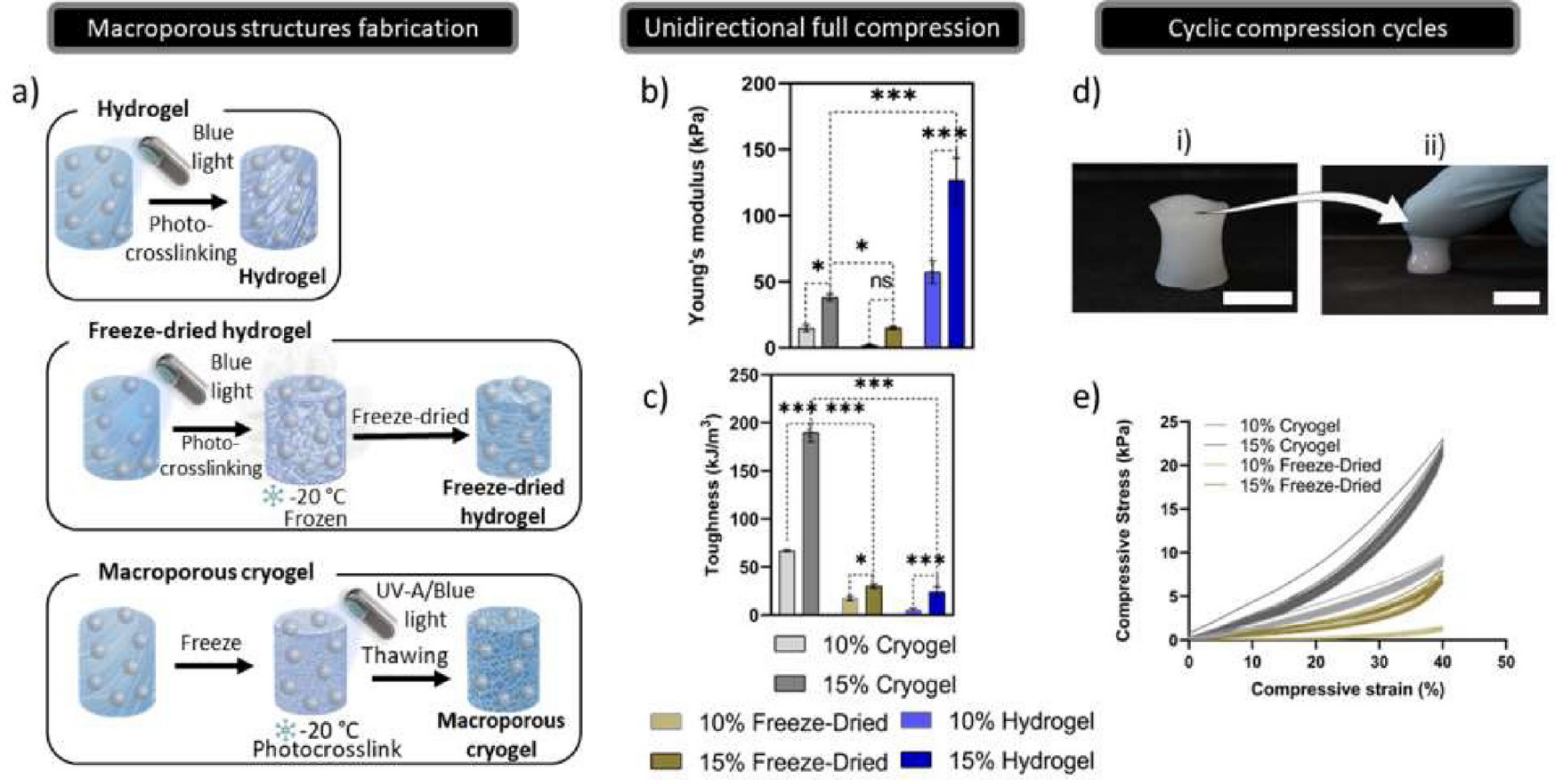
Mechanical properties assessment. a) Schematic representation for the different methodologies used for the production of hydrogels, freeze-dried hydrogels, and macroporous cryogels. b) Assessment of the different young modulus for each scaffold obtained from the slope in the first 5% of the strain/stress curves. c) Toughness values calculated through the area under the stress/strain curves for the different formulations. d) Cryogel compression without rupturing. Scale = 5 mm. e) 10 cycles of compressive load/unload stress for macroporous cryogels and freeze-dried hydrogels at 40% compression.

To generate MetLAM-MSNPs-CaP scaffolds, three different scaffolds were produced: hydrogels (Hs), freeze-dried hydrogels (FDHs), and macroporous cryogels (MCs). All scaffolds were casted in PDMS molds with 3 mm height and 5 mm of diameter. Hs were photopolymerized immediately after casting the solution into the PDMS molds. FDHs are a result of freeze-drying hydrogels, while for MCs, the solution on the PDMS molds was first frozen at −20 °C, followed by the photo-cross-linking process (Figure 2a). This way, an array of scaffolds with different types of porosity were envisioned: Hs with nanoporosity, FDHs with a low content of microporosity, and MCs with a higher micro- and macroporosity content. The multiple types of scaffolds fabricated were used to further optimize a method that can induce anisotropy and still afford good mechanical properties for injectability.

### Mechanical Assays

**Figure 3:**
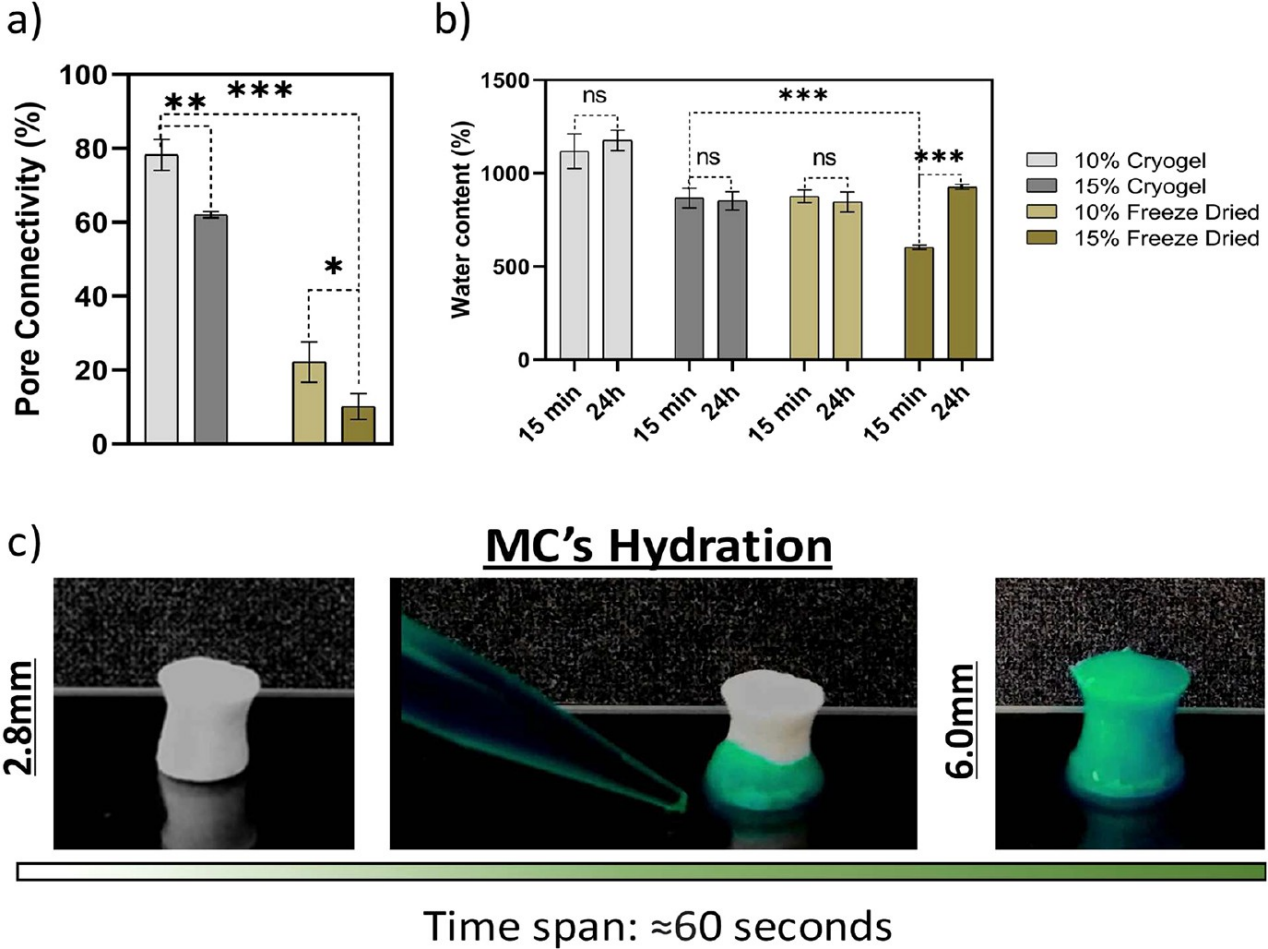
Swelling and pore connectivity. a) Quantification of pore connectivity obtained from an indirect assay to determine the pore volume for macroporous and freeze-dried hydrogels. b) Cryogel swelling in phosphate-buffered saline (PBS, pH 7.4) at two different time points: 15 min and 24 h. c) Time span captured by optical photographs showcasing the swelling of the 15% (w/v) macroporous cryogel.

To evaluate MCs mechanical properties, in comparison with the FDHs and Hs, full compression and cyclic compression tests were performed. Full compressive strains (Figure SI5) show that the MCs did not suffer any significant deformation until 95% strain. However, the FDHs did show permanent deformation at 85% and 80% for 10% and 15% (w/v), respectively. In comparison, Hs, which do not have a macroporous structure, ruptured around 40% and 45% for the same concentrations. Moreover, in general, the MCs (10% and 15% (w/v)) exhibit higher stiffness than the FDHs. On the other hand, Hs exhibit a substantially higher Young’s modulus (Figure 2b). This may be attributed to the nanoporous network and lack of void spaces that led to a compact scaffold. All the three types of scaffolds exhibited a higher Young’s modulus for the 15% (w/v) concentration. Although there are reports of softer cryogels enhancing the potential for injectability,^12^ they often lack in the stiffness required to generate a favorable environment for cells to differentiate into osteoblasts.^53,54^ Additionally, MCs (10% and 15%) displayed the highest toughness, 71 ± 7.6 and 190 ± 9.5 kJ/m^3^, respectively (Figure 2c). The MCs resilience can be visually observed (Figure 2d) where they can be easily squeezed to half their size without any indication of permanent damage to the structure. The dynamic stress–strain behavior of MCs and FDHs was then investigated to assess their high resilience, rapid recovery, and robustness, applying three distinct strains of 20%, 30%, and 40% for 10 cycles. Naturally, the strain of a defected bone tissue is exceptionally low and may vary between 18% and 34% of shear strain, accordingly to the distance to the defect.^55^ The data related to the 30% and 20% cyclic tests are presented in Figure SI6. After 10 cycles of 40% compressive strain, it is possible to observe that MCs show significant mechanical recovery at both concentrations, when compared with FDHs (Figure 2e). Loading and unloading data were also used to access the hysteresis loop and to calculate the dissipated energy involved in the deformation of the scaffold (Figure SI7a). Considering the first cycle, the energy dissipation obtained for the MCs was 1024 ± 119 and 396 ± 129 J/m^3^ for the 15% and 10% MetLAM, respectively. FDHs have shown energy dissipation of 369 ± 88 and 85 ± 40 J/m^3^, for the same concentrations. A possible explanation for the different values of dissipating energy during compressive stress is related to a higher pore content, which allows a greater energy dissipation toward the polysaccharide walls, while a compact matrix with less porosity builds up energy and leads to an easier mechanical rupture.^56^ MCs had a significantly higher energy hysteresis when compared with the FDHs, consistent with the range of values for energy hysteresis of previously reported cryogels.^15,57^ Besides, this effect is highly predominant during the first cycle, which is important for the determination of the cryogel’s injectable potential. The fact that the MCs can easily expel water through the interconnected pore network and quickly recover allows the scaffold to better withstand the compression forces and recover its shape upon release. As a result, MCs tend to be less susceptible to mechanical rupture, approximately 3-fold, when compared to the FDHs. It is noteworthy to point out that the MCs display a significantly better mechanical recovery (%) in both concentrations, 92.2 ± 2.1% and 88.6 ± 3.5% for 15% and 10% (w/v), respectively (Figure SI7b). When applying 40% compressive strain, no apparent recovery loss was observed and all scaffolds retained their shape and elasticity, indicating a strong compression resistance under significant compressive strains.^57^ Moreover, 15% MCs (w/v) were subjected to 100 cycles (at 30% compressive strain) to confirm its great robustness (Figure SI8 and Video SI1). In the video it is also possible to observe the shape-memory ability after each cycle: during the unloading, the MCs regain their original shape while taking up the surrounding water. This shows that the MCs present more of an elastic-like behavior than FDHs. Naturally, the presence of a higher dissipating energy will allow an easier and more efficiently material’s injectability, an important feature for the development of minimal invasive procedures. Moreover, SEM analysis was also used to further compare MCs and FDHs before and after being submitted to 10 cycles of loading–unloading at 40% compressive strain (Figure SI9a). Comparing both types of scaffolds, it is possible to see that overall MCs show a better recovery, with no significant deformation. On the other hand, the FDHs pores tend to be more squeezed and even appeared to be able to close upon different compression cycles. Thus, the pore distribution of both scaffolds at 15% (w/v) was assessed (Figure SI9b). It is possible to observe that MCs have a higher average of pore size (84 ± 50 μm) when compared with the FDHs (34 ± 18 μm) and a higher size polydispersity. Scaffold porosity plays a crucial function in the context of tissue regeneration, particularly in ensuring appropriate vascularization and, as a result, enhancing bone regeneration.^58^ It is known that the minimum porosity necessary for gas exchange and nutrient transport in the scaffold its around 30–40 μm.^59^ Thus, MCs have a higher tendency for improving nutrient mass transport compared to the FDHs which display lower pore sizes. Additionally, pore connectivity was determined (Figure 3a). As expected, there is a higher interconnected porosity for the MCs. For the 15% (w/v) formulations, the pore connectivity was around 62 ± 0.8%, still higher than the percentage found for the FDHs. Macroporous scaffolds have been reported to have an extremely fast swelling rate.^60,61^ After 15 min and 24 h of dried scaffolds being in contact with PBS, the water content was measured (Figure 3b). After 24 h, the water uptake had no significant differences in comparison with the 15 min time point. This was visually confirmed using dried MCs 15% (w/v), where it was possible to stabilize the water uptake in less than 1 min (Figure 3c and Video SI2), although it is possible to observe a swelling effect for the 15% (w/v) FDHs. MCs show a water content of 867 ± 54% for the 15% (w/v) concentration, while for the same FDHs concentration, the water content was 603 ± 11%. Remarkably, even though MCs have higher water content when compared with FDHs, they still show higher toughness and Young’s modulus. Thus, this method opens the possibility to have tougher scaffolds without compromising the cell growth and proliferation, since they are highly dependent on the overall water content and access to nutrients. Taking the aforementioned results into account, the 15% (w/v) MCs formulation was chosen for the following assays, since it showed the best mechanical properties for bone regeneration. MCs showed higher pore content, size, and connectivity while being more reproducible and having better recovery after compressive stress.

### Bioactivity Analysis

**Figure 4:**
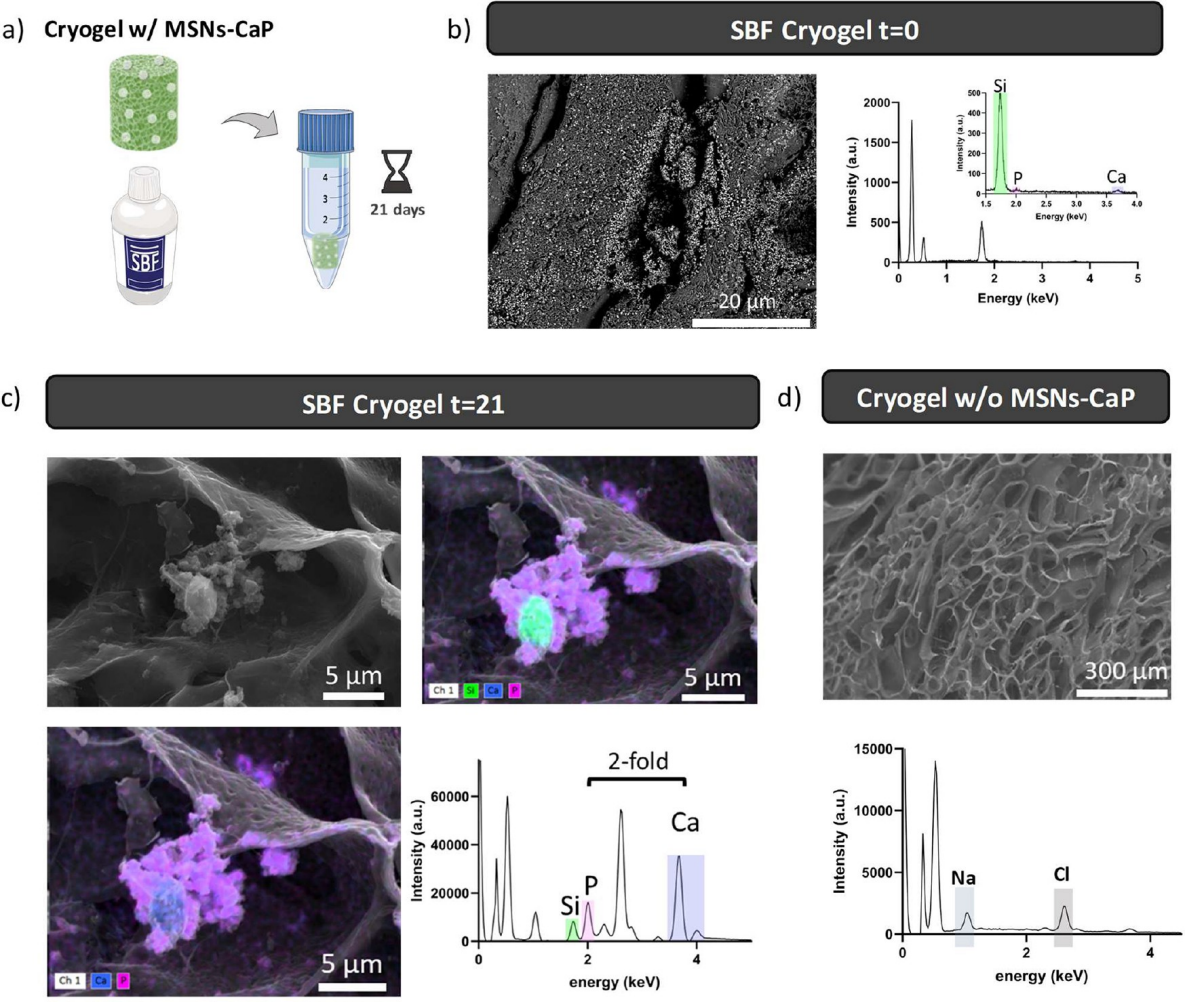
Simulated body fluid bioactivity of MSNPs-CaPs. a) Schematic representation for the MC with MSNPs-CaP on 21 days in SBF. b) SEM images of macroporous cryogels with MSNPs-CaP at day 0 of SBF andrespective EDS spectra; . c) SEM images of macroporous cryogels with MSNPs-CaP after 21 days in SBF at 37 °C highlighting the growth of calcium phosphate aggregate around a silica nucleus. Respective EDS spectra, elements were represented with different colors: silicon (green), phosphate (pink), and calcium (blue). d) SEM images of macroporous cryogels without MSNPs-CaP at day 21 of SBF and respective EDS spectra.

The bioactivity levels of MSNPs-CaP and MCs with MSNPs-CaP (B-MCs) were evaluated through 21 days of immersion in simulated body fluid (SBF) (Figures 4a and SI10). The growth of calcium phosphate aggregates was tracked using SEM, while the Ca and P ratio and overall content (wt %) were determined using SEM-EDS. After 21 days, the MSNPs-CaP were covered with needle-like nanohydroxyapatite crystals (Figure SI10, bottom). Additionally, by analyzing the EDS spectra, it is possible to observe the decrease of the silica peak intensity while the Ca and P peaks increased, indicative of hydroxyapatite mineralization (Figure SI10, right). Prior to the immersion in SBF, the percentages of Ca and P were 3.4% and 2.9%, respectively, and they increased to 29.6% and 15.0% after 21 days. The Ca and P molar ratio attained from the EDS spectra (1.97) is different from the theoretical value of 1.67. This can be explained by the fact that not all of the nanoparticle surface was covered with the nanohydroxyapatite needles; therefore, the background signal of MSNPs-CaP has influence in the overall EDS quantification. Despite the obtained value, ratios between 1 and 2 have proven to exhibit higher osteoblast viability and osteoblast alkaline phosphatase activity without stimulating the production of nitric oxides.^62^ After confirming that the silica MSNPs-CaP have the ability of promoting the growth of calcium phosphate aggregates, the same assay was carried out with B-MCs (15% (w/v) MetLAM loaded MSNPs-CaP). The overall Ca and P ratio and contents (element %) were measured at *t* = 0 (Figure 4b). After 21 days, it was possible to observe that B-MCs also promoted the growth of calcium phosphate aggregates on top of the cryogels (Figure 4c). The control (without MSNPs-CaP) did not show any signs of calcium phosphate aggregates after 21 days (Figure 4d). The efficiency of generating calcium phosphate aggregates with MSNPs-CaP was greatly proved. By using only 1% (w/v) nanoparticles, B-MCs were able to promote mineralization in the organic scaffold. The presence of calcium phosphate aggregates in B-MCs matrix is expected to contribute toward the bone-binding affinity.

### Rheology

**Figure 5:**
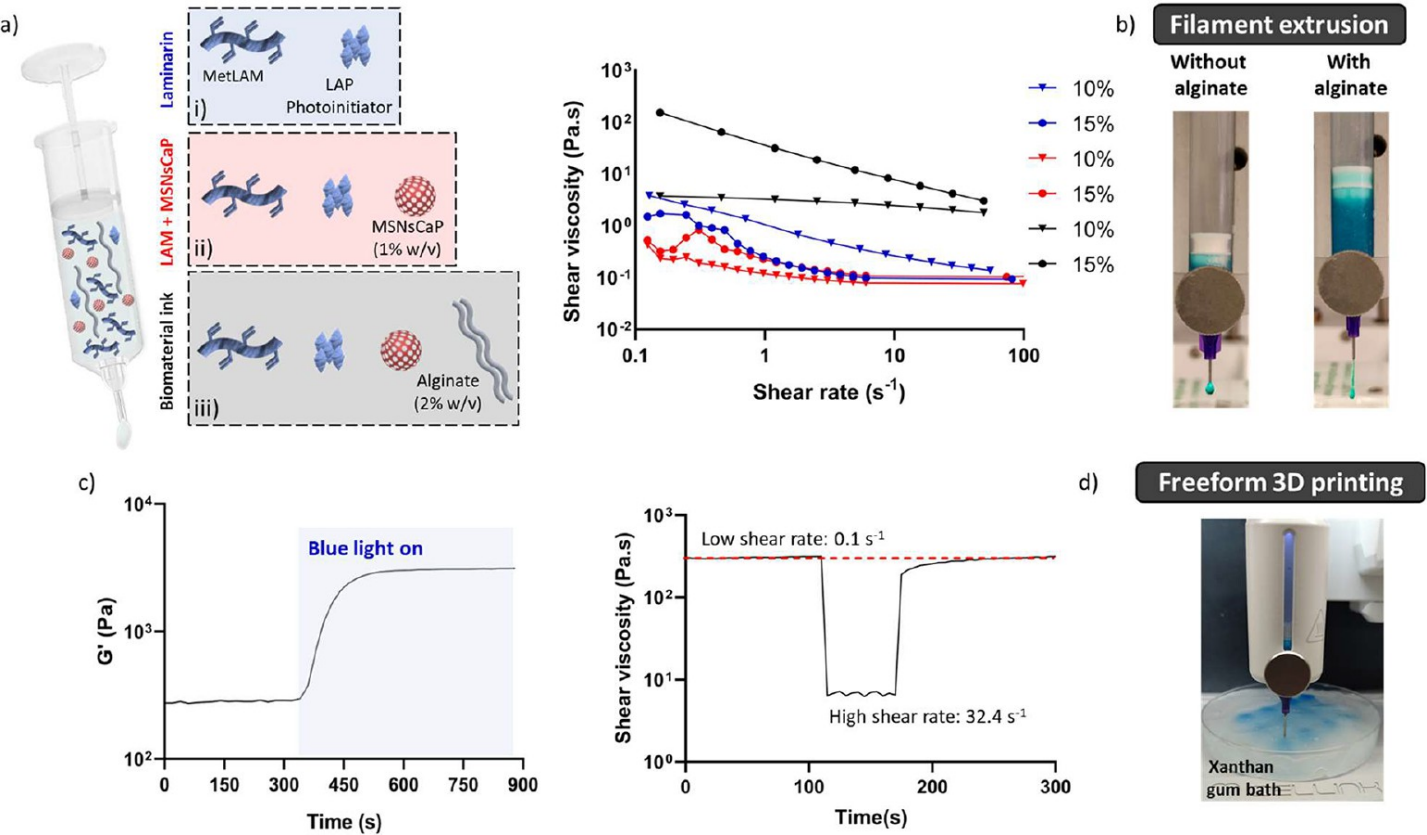
Rheology characterization of the LAM biomaterial ink. a) Schematic representation of the formulated biomaterial ink. b) Shear thinning characteristics of (i), (ii), and (iii) illustrated by shear viscosity vs shear rate. Photographic comparison between the filament extrusion of the (iii) and (ii). c) *In situ* photorheology data showcasing the storage modulus as a function of time for the biomaterial ink. Analysis of thixotropy for (iii). d) Printing process on a xanthan gum bath and blue colored biomaterial ink (iii).

A biomaterial ink composed of (i) MetLAM, (ii) alginate (as sacrificial component), (iii) photoinitiator, and (iv) bioactive MSNPs-CaP was screened for extrusion 3D printing. Rheological measurements were performed using 15% (w/v) MetLAM to access its potential to serve as a biomaterial ink and printing potential (Figure 5a). Alginate was used to increase the overall viscosity and to act as a sacrificial layer that has no chemical interactions with MetLAM or MSNPs-CaP. It was possible to observe that the biomaterial ink extruded without alginate is released as a droplet, while when biomaterial ink is mixed with alginate, a filament is achieved (Figure 5b, Video SI3). Hence, alginate not only increased the viscosity needed to stabilize the MSNPs-CaP but also led to a linear dependence of the shear rate. Moreover, with the addition of this highly viscous component, it was possible to stabilize the MSNPs-CaP deposition (Video SI4). The linear viscoelastic region (LVER) (Figure SI11) was determined by obtaining a linear region comprehended on the interval of shear strains values of 0.01–1.6%. The storage model (*G*′) increased immediately upon the application of UV-A/blue light with a full cross-linking after 3 min (Figure 5c). Thixotropy was evaluated to determine whether the microstructural arrangement could be preserved after extrusion. Thus, as indicated in eq 1 and through the power law index, obtained from the shear rate versus shear stress curves (Figure SI12), an experimental value of shear rate for the printing conditions was calculated. Using the calculated shear rate value of 32.44 s^–1^, a thixotropy assay was performed (Figure 5c). The initial viscosity of our precursor solution was ∼300 Pa·s, which decreased sharply to ∼7 Pa·s upon the application of a shear rate of 32.44 s^–1^. After removing the high shear rate, the viscosity recovers up to ∼300 Pa·s almost instantaneously, showcasing that the ink can rapidly recover to its initial structure. These results indicate that the extrusion process does not affect the structural integrity of the material.

### 3D Printing and Pore Analysis

**Figure 6:**
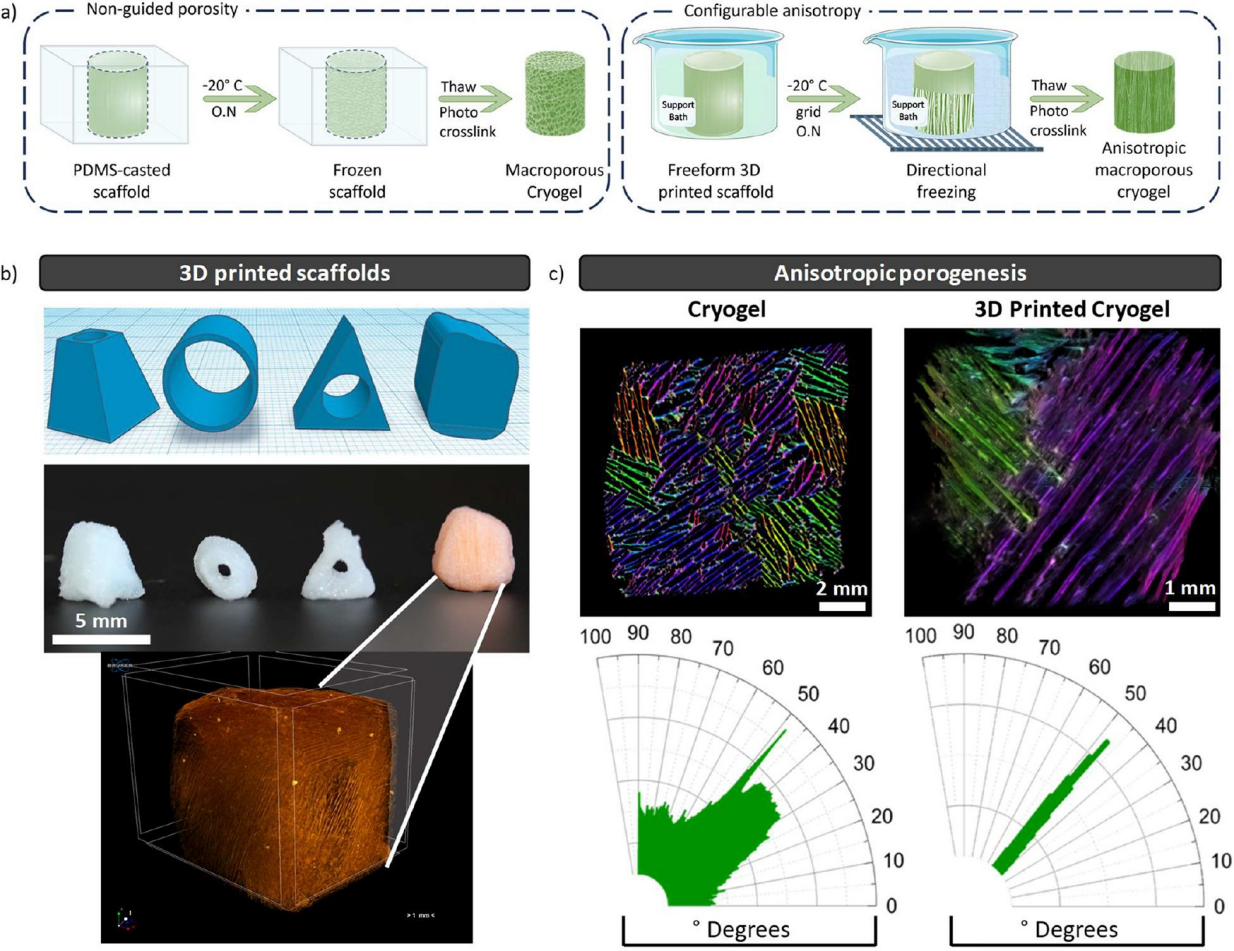
3D printing assisted with directional freezing. a) Schematic representation of the nonguided porosity (left) and configurable anisotropy (right) cryogel fabrication. b) 3D printed constructs: STL files (top); photo-cross-linked cryogels (center); 3D micro-CT image of the cubic anisotropic 3D printed cryogel (bottom). c) 3D micro-CT images of the anisotropic 3D printed cryogel and OrientationJ analysis with the respective color survey for the PDMS casted cryogel (control, left) and 3D printed with directional freezing cryogel (anisotropic, right). Some elements were adapted from the BioRender.com icons library.

3D printed cryogels for orthopedic applications have been shown to have several advantages. First and foremost, their injectability makes them highly versatile for minimally invasive procedures. Thus, it is possible to precisely deliver the cryogel to the site of the bone defect, conforming to its shape and size, without the need of extensive surgical procedures. This not only reduces patient discomfort but also facilitates targeted treatment, ensuring maximum efficacy. Furthermore, the ability to fabricate these cryogels from a model obtained through micro computed tomography (μ-CT) scans of the patient’s specific bone defect is ground-breaking. This personalized approach allows for the fabrication of cryogels that perfectly match the morphology of the defect, optimizing the integration and stability of the implant within the surrounding tissue. Hence, previous studies have shown that 3D printed cryogels that have the capability to completely fill the bone defect, are able to promote faster regeneration of the calvarial bone defects when compared to smaller or incomplete fillings.^63^ Herein, the biomaterial ink was freeform 3D printed in a viscoelastic continuous polymeric matrix of xanthan gum (Figure 5d). Multiple shaped structures were then kept inside the support bath and frozen at −20 °C. The resultant block of frozen xanthan gum and embedded scaffolds were fully photo-cross-linked with UV-A/blue light. After the constructs were thawed, they were easily retrieved from the support bath and washed with water. Thus, after cleaning the scaffolds with water, the alginate was easily removed and therefore considered as a sacrificial biopolymer. Suspension 3D printing allows the conjugation of a directional freezing procedure prior to cross-linking. Thus, simply by freezing the constructs embedded in a xanthan gum bath and holding it above a prechilled metal grid, a directional freezing front is generated (Figure 6a). This controlled freezing front generates an oriented growth of the ice crystals. The template expelled the polymer and particles present in the bath, restricting them in the interstitial space between the ice structures. Photo-cross-linking followed by thawing resulted in a highly aligned and hierarchical 3D porous material. Multiple shape-memory and free-standing nanocomposite structures were successfully fabricated in a short period of time (Figure 6b). Hence, the MCs fabricated with this method showed an oriented lamellar structure with a thickness of 19 ± 7 μm and an interlamellar space of 89 ± 16 μm (Figure SI13). The newly optimized directional ice templating is reproducible (*n* = 6), environmentally friendly, and possible to adjust to different methodologies.^64−66^ μCT was used to analyze the topographic porosity of the B-MCs cryogels casted in a PDMS mold (CB-MCs) and freeform 3D printed cryogels with directional freezing (PB-MCs) (Figure 6c, top). PB-MCs shown that 77% of the scaffold is comprised of pores whilst also presenting an extremely high value of interconnectivity (99.98%). ImageJ analysis was used to study the pore orientation of both scaffolds (Figure 6c, bottom).^730^ PB-MCs showed a very narrow distribution of pore orientation averaging at a 45° angle, while PC-MCs have no defined pore orientation with an interval of angles from 0° to 90°. The color survey for PB-MCs displays two distinct colors as the orientation is registered as −45° and 45° depending on *XYZ* point of view. Lamellae-oriented porosity obtained from directional freezing has already proved to enhance the osseous differentiation.^67^ Moreover, the resulting pore pattern may mimic bone porosity in size and shape, since larger osteons and vascular channels have around 100 μm of length and are found with similar orientation.^68^

### Cryogel Injectability

**Figure 7:**
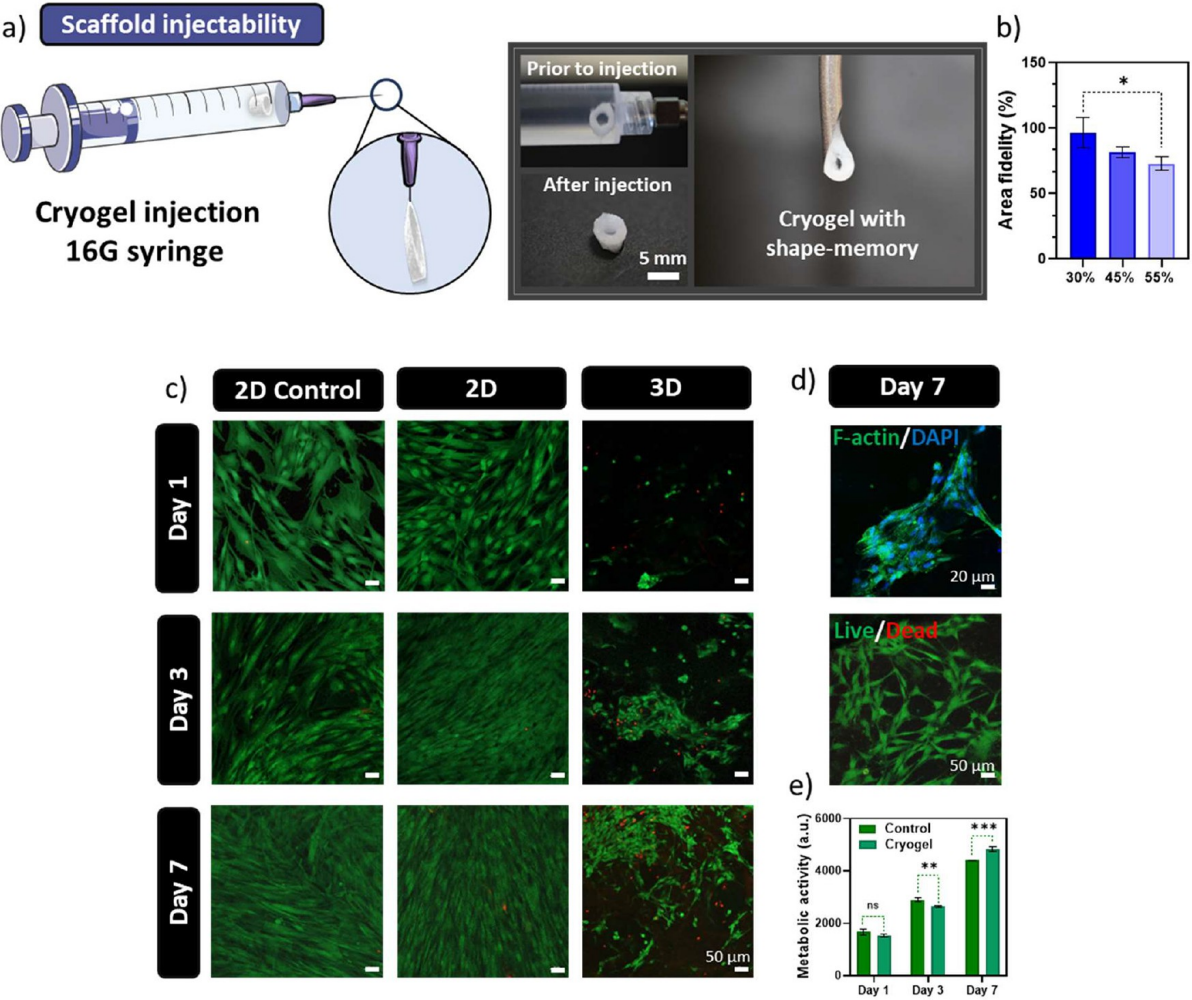
Injection and indirect cytotoxic assays. a) Schematic illustration of the 3D printed cryogel cylinders (left) and photographic time lapse cryogel loading inside a 16G syringe; demonstration of cryogel’s compression during and after injection (right). b) Cryogel compression and area fidelity upon injection of PB-MCs cubes. c) Live–dead micrographs of adhered hASCs (2D Control), adhered hASCs with PB-MCs (2D), and hASCs absorbed onto the PBMCs (3D), incubated for 1, 3, and 7 days; scale = 50 μm. Live cells, green channel (Calcein-AM). Dead cells, red channel (PI). d) F-actin (green) and DAPI (blue) micrographs of adhered cell on the PB-MCs (top); scale = 20 μm; Live–dead micrographs of released hASCs from the 3D culture onto a subtract (bottom); scale = 50 μm. e) hASCs metabolic activity using alamar assay after 1, 3, and 7 days.

For cryogels to flow through a conventional medical needle, it is important that they can support high shear stress and recover to the original structure without rupture (shape recovery). Thus, mechanically robust PB-MCs (hollow cylinders with 5 mm diameter and 5 mm of height) suspended in PBS were successfully injected through a 12-gauge syringe (∼40% compression) (Figure 7a). It is assumed that the total volume of the PB-MCs is able to be compressed over the pore void space and therefore squeeze through the needle’s gauge.^12^ Additionally, the ability to quickly expel and reabsorb water contributes to their injection. The robustness of the cryogels revealed a great volume stability and ability to return to the initial shape after deformation of injection. After three consecutive injections, there was no significant difference to their undeformed structure. During this procedure, the recovery time was too fast to be assessed, but immediately after injection, the scaffold appeared to be back to the original shape-fixed structure. The injection did not exhibit a significant loss of integrity even after the third injection. Moreover, to understand the maximum compressibility that the PB-MCs can withstand, different bulk cubes were printed. The cryogels were subsequently injected using a 12G needle up to a 18G needle (30%, 45%, and 55% compression). The total area was evaluated after each injection and compared to the initial total area (Figure 7b). The cryogel PB-MCs were able to withstand up to 45% compression without any significant signs of deformation (82 ± 4% total area). Although at 55% compression, the total area of the cryogel was reduced to a value of 73 ± 5%. By refining the in-fill printing parameters or incorporating hollow structures, the deformability of the cryogel can be further optimized, facilitating a seamless integration with the surrounding tissue and enhancing its utility. Such modifications are particularly advantageous considering that bigger structures will be possible to be injected, thus ensuring an effective interaction at the tissue interface with a high impact for cell-laden implants without the need of invasive techniques.^14,69^

### Cryogel Cytotoxicity

As a means to validate the cytocompatibility, human-derived adipose stem cells (hASCs) were used. hASCs were adhered to the surface of an Ibidi well plate. After 4 h the cryogel was placed on top of the adhered cells and remained up to 7 days (Figure 7c, 2D assay). Afterward, live–dead assays were conducted to assess the cell viability and compared to the control (cells were incubated without cryogels). As a result, no cytotoxic effect was observed as the cell proliferated until confluence without any signs of cellular death. Despite the absence of cell-adhesion motifs in PB-MCs, hASCs were successfully cultured within the large pores of the cryogel (Figure 7c, 3D assay). The dried cryogel was immersed in a suspension of hASCs to facilitate cellular absorption. Initially, a low cell density was observed; however, subsequent days revealed an increased proliferation of the hASCs. Furthermore, by day 7, substantial deposition of extracellular matrix within the cryogel structure was observed (Figure 7d, top). Notably, while cell growth occurred within the cryogel matrix, there was also evidence of hASCs release into the substrate (Figure 7d, bottom), suggesting the potential of PB-MCs as cell-delivery injectable biomaterial. Lastly, cellular metabolic activity was assessed using an alamar assay (Figure 7e). When compared with the control, hASCs metabolic activity had no significant alterations at the day 1; however, an increase at day 7 was observed. The cytotoxic results obtained for the PB-MCs serve as prominent indicators for prolonged cell culture periods. Hence, PB-MCs offer protection, sustenance, and a conducive microenvironment for post-transplantation cellular survival through minimal invasive techniques.

## Conclusion

Suspension 3D printable nanocomposite cryogels based on MetLAM, containing bioactive MSNPs-CaP and lamellar topology, were successfully developed for the first time. The formulated biomaterial’s ink demonstrated a shear-thinning behavior, printability, and outstanding mechanical performance of the scaffolds such as shape memory and injectability. In this study, there was an extensive characterization of the mechanical properties comparing cryogels with freeze-dried hydrogels and conventional hydrogels. Mainly, the nanocomposite cryogels have shown the ability to withstand full compression without rupture while maintaining their mechanical recovery. The scaffolds preserved their structural integrity and shape-fixed structure upon injection with an extremely fast recovery time. The cryogels also showed a larger and fully interconnected macroporous network when compared to bulk hydrogels and FDHs. Furthermore, the materials have proven to be composed of bioactive nanoparticles and noncytotoxic effects toward human-derived adipose stem cells. Thus, a promising cell-delivery cryogel was produced herein for minimally invasive applications. The ability to encapsulate and protect hASCs provides a conducive environment for cell proliferation, differentiation, and tissue regeneration. The lamellar topography with a narrow orientation distribution was achieved via a simple method without the need for typical copper supports or liquid nitrogen temperatures. Thus, herein we optimized a straightforward method of producing injectable 3D printed nanocomposite cryogels with the engraving of a lamellar topology through a directional freezing method. The 3D printed PB-MCs cryogels herein developed represent a promising avenue for bone regeneration due to their injectability, patient-specific fabrication, ability to completely fill defects, and capacity to serve as cell delivery biomaterials with the expected bone-binding affinity. Future studies must be performed in conjunction with cell active materials in order to explore the potential effects of the lamellar topography and osteogenesis.

## Experimental Methods

### Materials

Laminarin (LAM) from *Eisenia bicyclis* (CAS: 9008–22–4) and lithium phenyl(2,4,6-trimethylbenzoyl)phosphinate (LAP) were purchased from Biosynth. Tetraethyl orthosilicate (TEOS, 98%), hexadecyltrimethylammonium bromide (CTAB, 98%), calcium chloride anhydrous, dry dimethyl sulfoxide (DMSO, p.a. ≤ 0.02% water), and formaldehyde were acquired from Sigma-Aldrich. 3-(Trimethoxysilyl)propyl acrylate (stabilized with BHT) (TMOS-PA, 93%) and glycidyl methacrylate (stabilized with MEHQ, 95%) were purchased from TCI Chemicals. Disodium hydrogen phosphate monohydrate and alginic acid sodium salt (*M*_w_ 10,000–600 000 Da, Alginate) were bought from PanReac AppliChem. 4-Dimethylaminopyridine (DMAP, 99%) and toluene (extra dry, 99.85%) were obtained from ACROS Organics. Unless otherwise specified, all chemicals were used as received without further purification.

### Synthesis and Characterization of Methacrylated Laminarin (MetLAM)

MetLAM was synthesized as previously reported by our group.^39^ Briefly, 1.0 g (5.56 mmol of glucose units) of LAM was dissolved in 10.0 mL of dry DMSO under a N_2_ atmosphere. To this mixture was first added 167.0 mg (1.36 mmol) of DMAP, following with the addition of 56.9 mg (0.4 mmol) of glycidyl methacrylate. The reaction mixture was stirred at room temperature for 48 h, and then the reaction was stopped by adding an equimolar amount of HCl solution (37% v/v). The modified LAM was purified by dialysis using a dialysis membrane with a molecular weight cutoff (MWCO) of 3.5 kDa against distilled water. Purified MetLAM was obtained by freeze-drying, giving rise to a white foam. The chemical modification was confirmed by ^1^H NMR and ATR-FTIR. The DS was calculated by ^1^H NMR as already reported.^39^^1^H NMR spectra were recorded on a Bruker Avance II 300 spectrometer (300.13 MHz; Bruker, Germany) using D_2_O as the internal reference and Mnova NMR (Mestrelab Research, S.L.) to analyze the data. The ATR-FTIR spectra of LAM and MetLAM were acquired in the absorbance mode by using a Bruker TENSOR 27 FTIR spectrometer (Thermo Scientific, USA) fitted with a “Golden Gate” ATR module with a diamond crystal. The ATR-FTIR spectra were measured in the spectral range of 4000–400 cm^–1^ by averaging 256 individual scans per sample, at a resolution of 4 cm^–1^. All data were linear baseline corrected and normalized using the OPUS software supplied with the instrument.

### Synthesis and Characterization of MSNPs Doped with Calcium and Phosphate

MSNPs synthesis was performed by an adapted sol–gel procedure described in the literature.^48^ Briefly, in a 500 mL polypropylene flask, Milli-Q water (240 mL), CTAB (0.5 g), and 1.7 M NaOH solution (1.75 mL) were added. The solution was stirred at 30 °C, until the temperature inside was stable at 30 °C. After 30 min, 21% (w/v) of anhydrous calcium chloride and disodium hydrogen phosphate monohydrate, in a 5:3 ratio, respectively, was added to the solution. After 30 min, TEOS (2.5 mL) was added dropwise, and the reaction mixture was left stirring for 2 h. After this time, the particles were recovered by centrifugation and washed three times with ethanol at 20000 *g* for 20 min at room temperature. Particle precipitate was dried at 50 °C overnight and afterward vacuum-dried to obtain a white powder. Consequently, to remove the surfactant template, the particles were calcinated at 600 °C for 4 h. TEM micrographs were acquired with a JEOL HR-(EF) TEM 2200FS, and the overall size and particle distribution was analyzed using ImageJ (*n* = 50). DLS measurements were performed on a Malvern instrument, Zetasizer Nano ZS, with filtered (0.45 μm PP filter) ethanolic solutions (0.5 mg/mL of MSNPs-CaP). The N_2_ adsorption/desorption isotherms were measured at 77.3 K using a Micromeritics Instrument Corp. Gemini V-2380. To functionalize the nanoparticle surface with acrylate groups, particles were dispersed in dry toluene (5 mL per 0.1 g of particles) and sonicated for 20 min. To the particle suspension was dropwise added 40 μL of TMOS-PA, and the sample was refluxed for 24 h under a nitrogen atmosphere. TMOS-PA volumes were calculated based on a target surface coverage of 3 molecules/nm^2^ and a particle density of 0.34 g/cm^3^. Particles were then recovered by centrifugation and washed three times with ethanol at 15000 *g* for 10 min at room temperature. Particle precipitate was dried at 50 °C overnight and afterward vacuum-dried to obtain a white powder. Quantification was performed by ^1^H NMR in D_2_O (pH = 13) using trioxane as an internal standard, through a procedure previously reported.^49^

### Cast Cryogels Preparation

To carry out (i) mechanical and (ii) bioactivity assays, the cryogels were prepared in PDMS molds (5 mm diameter and 3 mm height). The mold was frozen at −20 °C overnight, with subsequent photopolymerization resorting to a UV-A/blue light (λ = 385–515 nm Valo grand, 3 min with 1000 mW/cm^2^). To perform the comparative studies, freeze-dried sponges were also prepared, using the same formulations, but after the transfer to PDMS molds the solution was photopolymerized, frozen at −20 °C overnight, and freeze-dried for 24 h. Hydrogel were photopolymerized immediately after casting the solution into the PDMS molds. Prior to any assessment, the scaffolds were kept in PBS for 24 h.

### Mechanical Analysis

Using a Universal Mechanical Testing Machine (Shimadzu MMT-101N, Shimadzu Scientific Instruments, Kyoto, Japan) equipped with a load cell of 100 N, the mechanical behavior of hydrogel, freeze-dried, and cryogel scaffolds were evaluated with compression tests. Both unidirectional and cyclic compression assays were performed at room temperature on freshly hydrated cylindrical specimens with a diameter of 5 mm and height of 3 mm at a constant rate of 3 mm min^–1^. The Young’s modulus was defined as the slope of the linear region of the strain–stress curve, corresponding to a maximum of 5% strain. For the dynamic strain–stress behavior, the recovery percentage was calculated taking the compressive stress (kPa) of the first cycle as 100%. The 10 cycles dynamic strain–stress test were performed at 40, 30, and 20% compressive strain. For the 100 cycle test, 30% compressive strain was used. The toughness was calculated as the area under the stress–strain curves. Dissipation of energy was calculated by the hysteresis loop, represented by the area enclosed within the loading and unloading curve, as the amount of mechanical energy dissipated.^56^

### Scanning Electron Microscopy (SEM)

SEM was also employed to evaluate the morphology of the scaffolds. First the specimens were dried in an ethanol gradient (10, 30, 50, 80, and 100%) and left to dry overnight at room temperature. Next, the specimens were fixed on a metal stub using double sided adhesive carbon tape (Agar Scientific, AGG3939), sputtered with gold/carbon, and analyzed in a Hitachi SU-3800 coupled with a Brunker Quantax compact 30 detector. To assess the bioactivity assay results with the MSNPs-CaP, the particles were first deposited on a metal stub using an ethanol dispersion (0.1 mg/mL) and then left to dry for 1 day at room temperature. The sample was then sputter-coated with gold and analyzed with a Hitachi SU-70 SEM microscope.

### Water Content and Pore Connectivity

To evaluate the swelling ratio of cryogels and freeze-dried scaffolds (*n* = 6), the specimens were freshly prepared, freeze-dried, and subsequently immersed in PBS for 15 min and 24 h. The swelling ratio (*Q*_m_) was calculated by the following equation:
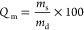
1where *m*_s_ and *m*_d_ are the weight of swollen and dried gels, respectively. The pore connectivity was assessed using a water wicking technique previously reported.^12^ Briefly, the interconnected porosity was calculated as the leaked volume over the total volume. The leaked volume is defined as the water mass that was wicked away using Kimwipe paper after the specimens were soaked in water for 1 h (total volume).

### Bioactivity Assay

To study the in vitro bioactivity of MSNPs-CaP, the samples were immersed in simulated body fluid (SBF) at 37 °C under orbital shaking (50 rpm). The preparation of SBF followed the protocol described by Kokubo.^70^ Briefly, 5.0 mg of MSNPs-CaP was immersed in 4.0 mL of SBF for 7, 14, and 21 days. After removing the samples from SBF, they were washed by centrifuging with ultrapure Milli-Q water (three times at 5000 *g* for 15 min) and dried at 50 °C for 3 days. Cryogels (cylinders with 5 mm diameter and 3 mm height) were also immersed at the same time points in SBF. Furthermore, the cryogels were carefully washed with Milli-Q water and left to dry at room temperature for 3 days. The samples were then analyzed by SEM coupled with EDS (Hitachi SU-3800 coupled with a Brunker Quantax compact 30 detector).

### Rheological Analysis

A Kinexus Lab+ (Malvern Panalytical, UK) was used to characterize the rheological properties of the materials. The gap setting was fixed at 0.5 mm, the tests were performed at 25 °C with a frequency of 1.0 Hz, and the shear strain was fixed between 0.01 and 1.6% (LVER). The elastic and viscous moduli of the precursor solution during the photopolymerization process was studied by photorheology resorting to a UV-A/blue light (395–480 nm Valo grand). Additionally, the shear-thinning behavior of the material was studied resorting to the analysis of the shear viscosity (Pa·s) while the shear rate (s^–1^) increased. The thixotropy features of the material were also studied. This test is related to the time needed for the microstructural rearrangement that occurs in a shear-thinning fluid, following an applied shear rate. In this case, the extrusion shear rate used for the thixotropy was selected according to predefined printing conditions. To determine that value, we performed a shear rate versus shear stress test, and the data was fitted to the power law equation (eq 2) to calculate the power law index (*n*)^71,72^
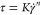
2where τ is the shear stress, γ is the shear rate, *n* is the power law index, and *K* is the consistency index. From this, the power law index was calculated and used in eq 3 to calculate the shear rate at the printer nozzle.
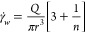
3where γ̇*˙*_*w*_ is the shear rate at the wall of the nozzle, *Q* is the volumetric flow rate through the nozzle, and *r* is the nozzle inner radius.^72^

### Biomaterial Ink Preparation

The biomaterial ink was prepared by first dissolving MetLAM 15% (w/v) in double distilled and deionized filtered (0.22 μm, 8.2 MΩ·cm at 25 °C) (Milli-Q) water. To this solution was added 1% (w/v) of MSNPs-CaP powder, and the mixture was placed in an ultrasonic bath (Elma, Elmasonic P, at 40 °C) for 30 min to full disperse the silica nanoparticles. After this, 0.5% (w/v) of LAP (the photoinitiatior) and 2% (w/v) of alginate were added to improve the printability.

### 3D Printing

The 3D solid structures were designed with SolidWorks software and then exported to the STL format. The cryogels structures with assorted sizes and geometries were 3D printed using a 23G nozzle (inner diameter = 337 μm). The printing process was performed at room temperature, with an extrusion pressure of 60 kPa and a needle speed of 100 mm·s^–1^ using an Inkredible+ 3D Bioprinter (Cellink, Gothenburg, Sweden) into a support bath of xanthan gum (5% (w/v)). Then, the 3D printed constructs were frozen overnight at −20 °C (with and without support of a prechilled metal grid) within the supporting bath, followed by photopolymerization resorting to a UV-A/blue light (λ = 385–515 nm, Valo grand, 3 min at 1000 mW/cm^2^).

### Micro-CT Analysis

The samples (15% (w/v) cryogels) were analyzed in an X-ray microcomputed tomography (μCT) SkyScan 1275 equipment (Bruker μCT, Kontich, Belgium) with penetrative X-rays of 30 kV and operated at 125 μA in high-resolution mode with a pixel size of 8 μm and 450 ms of exposure time. NRecon (Bruker, Kontich, Belgium), DataViewer (Bruker, Belgium), and CTVox software (Bruker, Kontich, Belgium) were implemented to create and display reconstructed results on the three orthogonal projections and 3D reconstructions, respectively. The analysis of the pore orientation was performed resorting to OrientationJ plugin from ImageJ.^730^

### Injectability Evaluation

Freeform 3D printed PB-MCs cubes with different sizes (*l* = 2.5–4.0 mm) were used to study the shape recoverability and maximum compression. The cryogels were subsequently injected through different gauge needles: 12, 14, 16, and 18G. The shape fidelity was observed under a magnifying microscope before and after every injection, and the area was evaluated using ImageJ.

### Cell Culture

To assess the *in vitro* biological performance, human-derived adipose tissue derived mesenchymal stem cells (hASCs, ATCC PCS-500–011) were used. Cells were seeded in a tissue culture flask T-175 cm^2^ (Sarstedt) using α-MEM under a controlled atmosphere of 5% CO_2_ and 37 °C. Upon reaching confluence, cells were trypsinized and seeded in an adherent 8-well Ibidi slide (live/dead) or an adherent 96-well plate (alamar). The PB-MCs were placed in contact with the adhered hASCs after 4 h. For the 3D cytotoxic procedure, the scaffolds were then brought in contact with the hASCs suspension for 4 h and afterward transferred to a different well with fresh medium, where they were cultured for up to 7 days.

### Cell Cytotoxicity

Briefly, after 1, 3, and 7 days the cells were incubated with calcein-AM/propidium iodide (PI) (Live–Dead Kit, ThermoFischer Scientific) for 20 min, according to the manufacturer’s protocol. The images were acquired with a confocal laser scanning microscope (CLSM 900, Carl Zeiss Microscopy, Germany) equipped with a 10× objective. For staining of F-actin filaments and nuclei, the cryogels containing cells were washed with PBS and fixed in 4% (w/v) formaldehyde for 20 min at room temperature. Before staining, the cells were permeabilized for 5 min with a 0.1% solution of Triton X-100. After being washed three times with PBS, samples were first incubated in a phalloidin solution (Flash Phalloidin Green 488) diluted 1:40 in PBS at room temperature for 90 min. After the gels were washed once with PBS, a DAPI solution (5 μg/mL) solution diluted 1:200 in PBS was prepared and used to incubate the cryogels for 5 min at room temperature. After several PBS washes, samples were visualized with a confocal laser scanning microscope (CLSM 900, Carl Zeiss Microscopy, Germany) equipped with a 25× objective. Acquired data was processed in Zeiss ZEN v2.3 blue edition software.

### Cell Metabolic Activity Quantification

To evaluate cell metabolic activity, alamarBlue Cell Viability Reagent (Alfagene, Lisbon, Portugal) was used. Briefly, the cell culture medium was removed, and 10% (v/v) solution of Alamar in α-MEM was added and left to react for 4 h at 37 °C. The results were obtained by measuring the absorbance at λ = excitation/emission (570/585 nm) (Microplate Reader Synergy HTX with luminescence, fluorescence, and absorbance, Biotek Instruments, Winooski, VT).

### Statistical Analysis

All data were statistically evaluated resorting GraphPad 8.0.1 software and reported as mean ± standard deviation (*n* = 3). Statistical differences were considered significant when the *p*-value < 0.05, established by unpaired *t* test.

## Supporting Information Available

FTIR analysis for GelMA modification; BET pore size, dynamic light scattering and energy dispersive analysis for MSNPs-CaP; compressive curves, recovery (%) and energy dissipation for cast scaffolds; scanning electron microscopy and energy dispersive analysis for scaffold’s pore size and bioactivity assessment; rheological properties of the biomaterial ink; scanning electron microscopy of cryogel anisotropy (PDF)
Videos SI1–SI4, showing uniaxial compression stress strain (100 cycles), cryogel swelling, 3D bioprinter filament extrusion, and MSNPs-CaP stabilization on the biomaterial ink, respectively (ZIP)
