# Supplementary material for: In-Bath 3D Printing of Anisotropic Shape-Memory Cryogels Functionalized with Bone-Bioactive Nanoparticles
Source: ACS Appl Mater Interfaces. 2024 Apr 9;16(15):18386–99. doi: 10.1021/acsami.3c18290 (PMC11040583; doi:10.1021/acsami.3c18290)
Supplement: Supplementary file 1 — am3c18290_si_001.pdf [file am3c18290_si_001.pdf]

Supporting Information

In-bath 3D Printing Anisotropic Shape-Memory Cryogels Functionalized with Bone Bioactive Nanoparticles

Edgar J. Castanheira‡, Luís P. Monteiro‡, Vítor M. Gaspar, Tiago R. Correia, João M. M. Rodrigues*, João F. Mano*

CICECO – Aveiro Institute of Materials, Department of Chemistry, University of Aveiro, 3810-193 Aveiro, Portugal

‡These authors contributed equally for this work.

* Correspondence and requests for materials should be addressed to: João M. M. Rodrigues (jrodrigues@ua.pt) and João F. Mano (jmano@ua.pt).

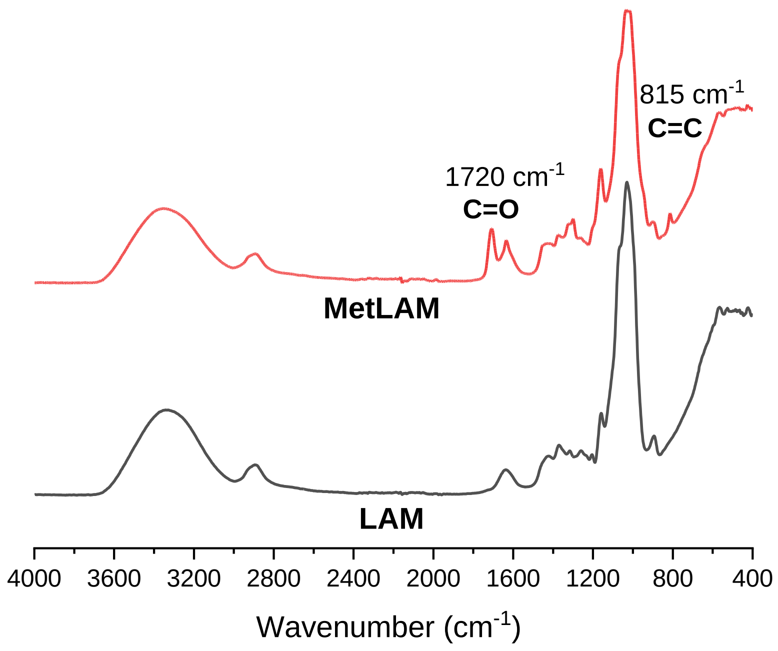

Figure SI1 – FTIR analysis of the biomaterial ink precursors. LAM (unmodified); Met-LAM (modified with glycidyl methacrylate).

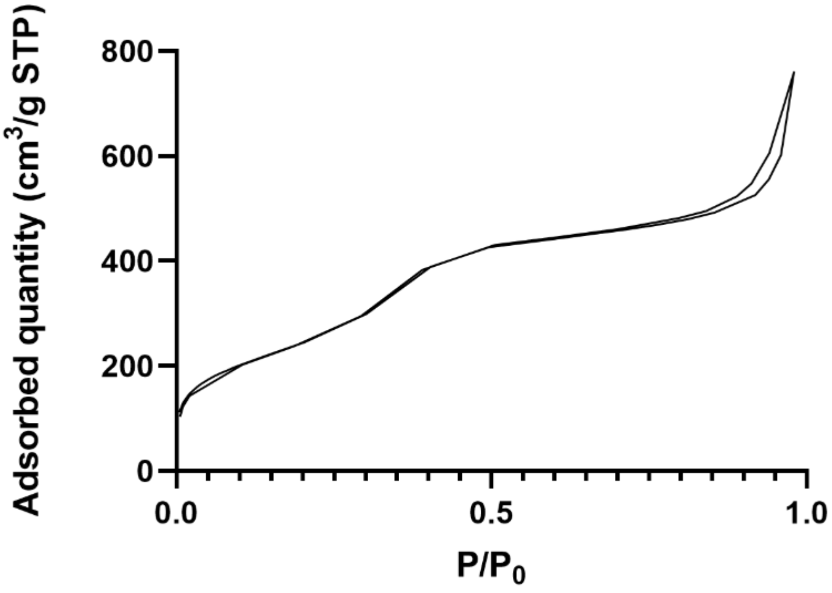

Figure SI2 – BET nitrogen adsorption isotherm plot analysis for determining the pore size of the mesoporous silica nanoparticles doped with calcium and phosphate.

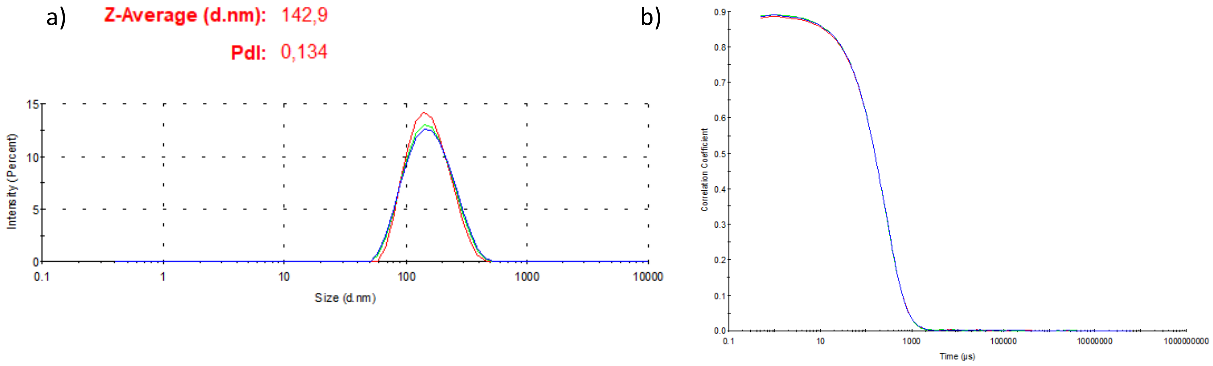

Figure SI3 – a) NPs size and distribution obtained by dynamic light scattering (DLS). b) Correlation curve of the respective DLS data presented in a).

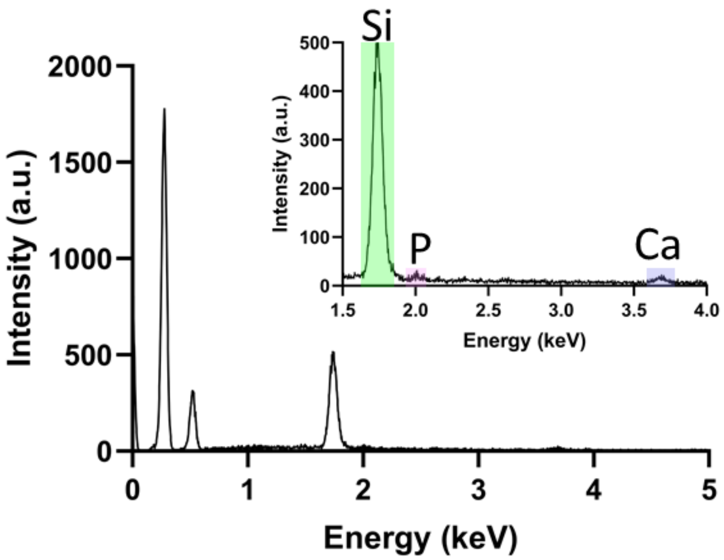

Figure SI4 – EDS spectra of the MSNPs-CaP after synthesis with a zoomed section to highlight the three elements: Si, Ca and P.

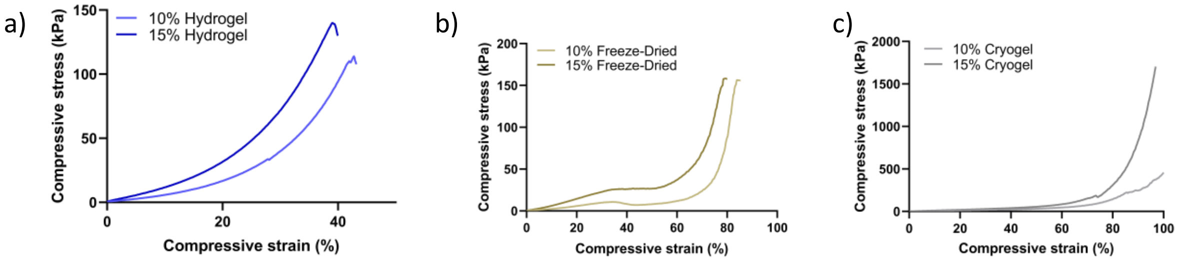

Figure SI5 – Full compressive unidirectional strain/stress curves for 10% (w/v) and 15% (w/v) a) hydrogels, b) freeze-dried hydrogels and c) macroporous cryogels.

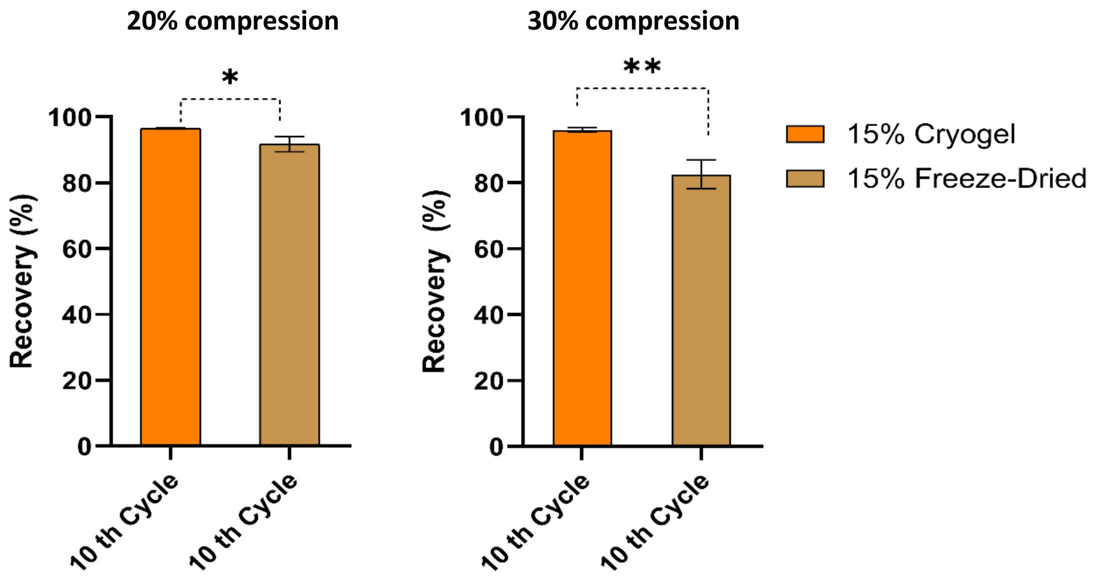

Figure SI6 – Recovery for the macroporous cryogel and the freeze-dried hydrogel at 15% (w/v) MetLAM and 1% (w/v) MSNPs-CaP between the first and tenth cycle loading–unloading hysteresis curve at 20% (left) and 30% (right) compression.

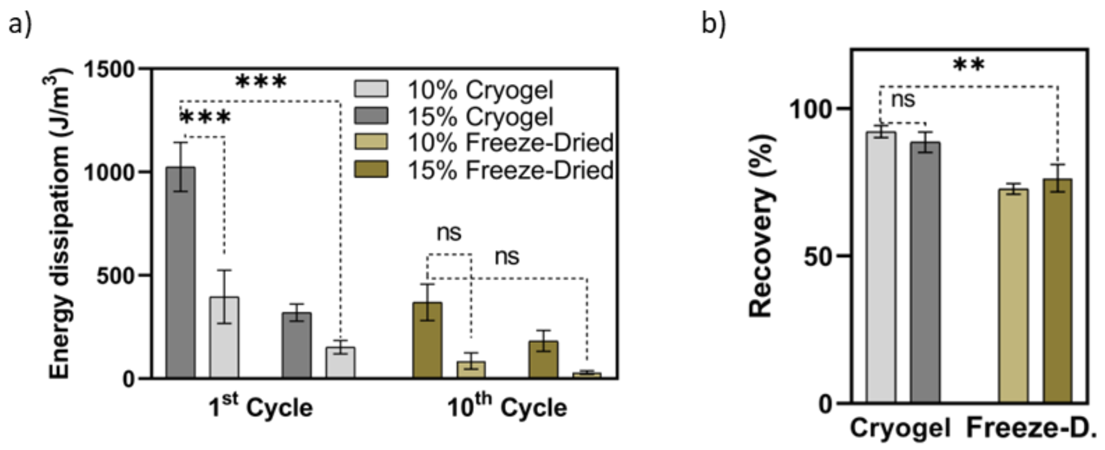

Figure SI7 – a) Dissipation energy during hysteresis of cryogels calculated from the loop area in the loading–unloading hysteresis curve during different compressive cycles at 40% compression and comparison between the first and tenth cycle recovery. b) Recovery (%) calculated from the loading-unloading hysteresis curve during different compressive cycles at 40% compression and comparison between the first and tenth cycle recover.

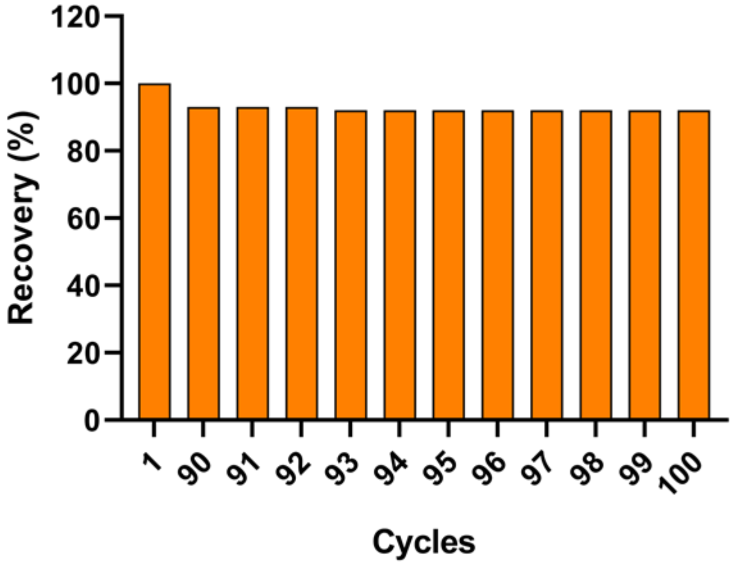

Figure SI8 – Recovery for the macroporous cryogel at 15% (w/v) MetLAM and 1% (w/v) MSNPs-CaP between the first and last ten cycles of 100 loading–unloading hysteresis curves at 30% compression.

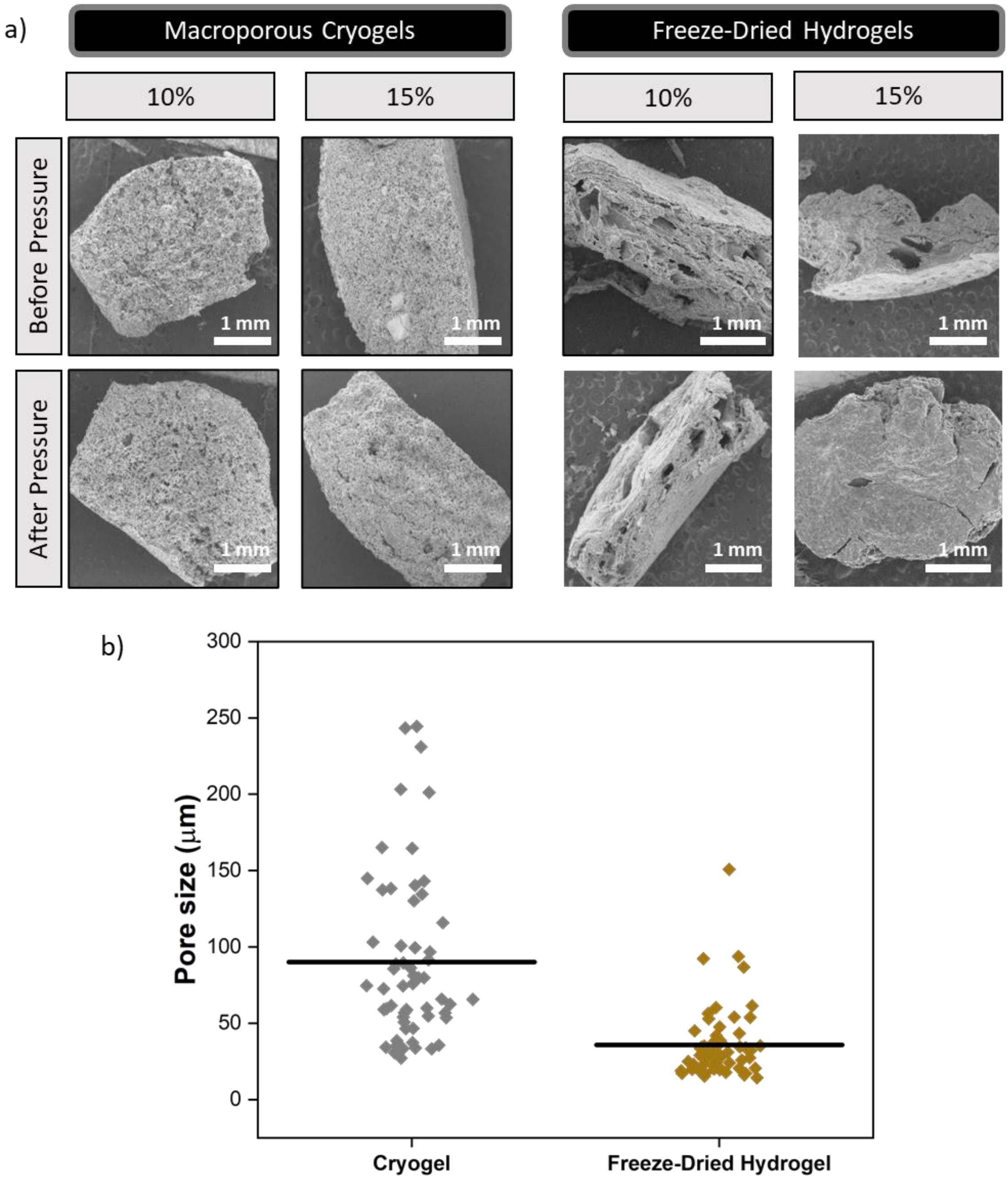

Figure SI9 - a) SEM morphology analysis of both cryogels before and after ten load/unloading cycles of 40% compressive strain; b) Pore size scatter analysis of 15% (w/v) MetLAM macroporous cryogels and freeze-dried hydrogels, black line shows the mean value.

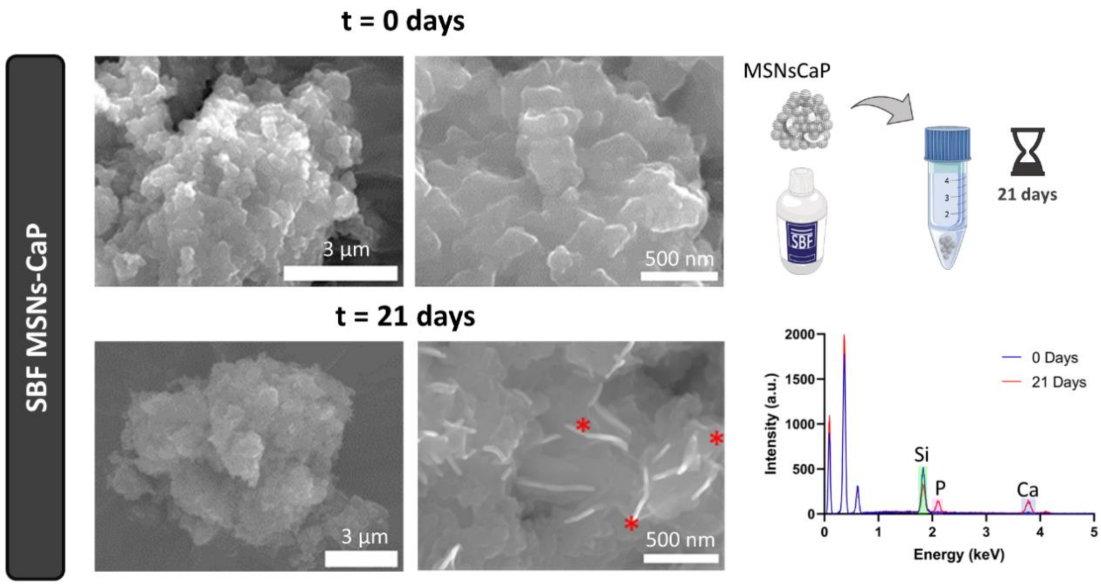

Figure SI10 - Simulated body fluid - bioactivity of MSNPs-CaPs. SEM pictures of the MSNPs-CaP immersed in simulated body fluid at day 0 and 21 – rod shaped hydroxyapatite crystals formation was highlighted with red asterisk. Respective EDS spectra highlighting the overall differences in intensities at the two different timepoints. Elements were represented with different colors: silicon (green), phosphate (pink) and calcium (blue).

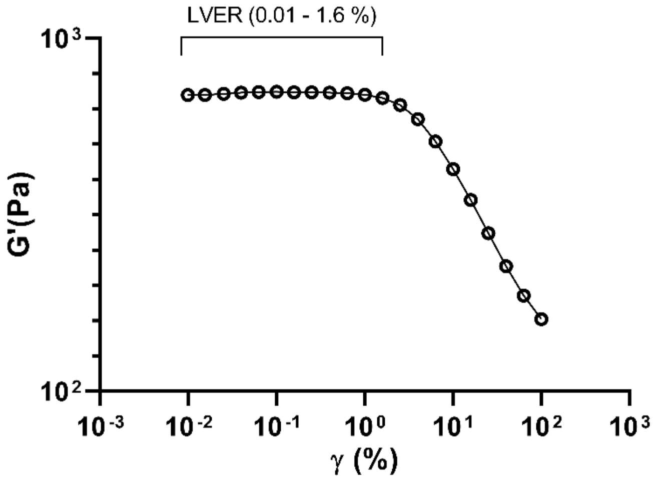

Figure SI11 – Strain sweeps ranging from 0.01 to 100% to determine the LVER (using the “plateau” or linear portion of the moduli curves), for the 15% (w/v) MetLAM and 1% (w/v) MSNPs-CaP biomaterial ink at 25 °C.

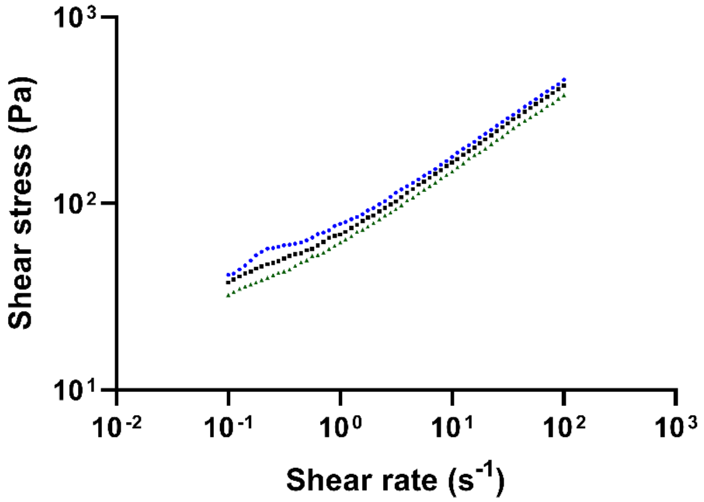

Figure SI12 – Shear rate *vs.* shear stress curves, at 25 °C, for MetLAM biomaterial ink at a concentration of 15% (w/v) LAM and 1% (w/v) MSNPs-CaP (*n*=3).

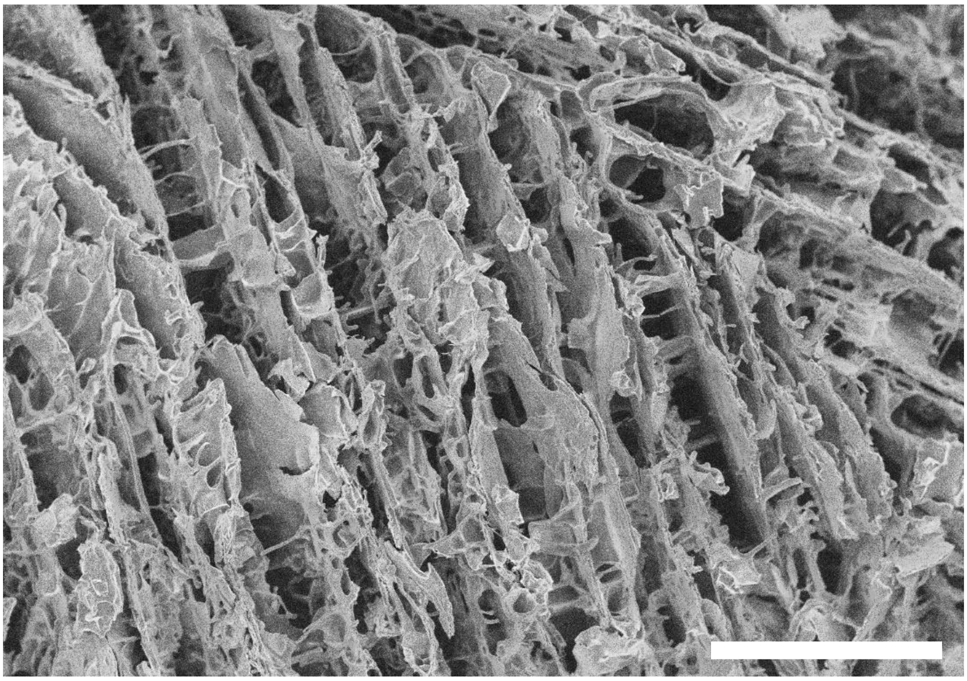

Figure SI13 – SEM image of fabricated PB-MCs demonstrating an oriented lamellar structure. Scale bar = 500 µm.
